# Aldo-keto reductase 1C1 induced by interleukin-1β mediates the invasive potential and drug resistance of metastatic bladder cancer cells

**DOI:** 10.1038/srep34625

**Published:** 2016-10-04

**Authors:** Ryuji Matsumoto, Masumi Tsuda, Kazuhiko Yoshida, Mishie Tanino, Taichi Kimura, Hiroshi Nishihara, Takashige Abe, Nobuo Shinohara, Katsuya Nonomura, Shinya Tanaka

**Affiliations:** 1 Department of Cancer Pathology, Hokkaido University Graduate School of Medicine, N15, W7, Kita-ku, Sapporo 060-8638, Japan; 2 Department of Renal and Genitourinary Surgery, Hokkaido University Graduate School of Medicine, N15, W7, Kita-ku, Sapporo 060-8638, Japan; 3 Department of Urology, Tokyo Women’s University Hospital, Shinjuku-ku, Japan; 4 Department of Translational Pathology, Hokkaido University Graduate School of Medicine, N15, W7, Kita-ku, Sapporo 060-8638, Japan

## Abstract

In treating bladder cancer, determining the molecular mechanisms of tumor invasion, metastasis, and drug resistance are urgent to improving long-term patient survival. One of the metabolic enzymes, aldo-keto reductase 1C1 (AKR1C1), plays an essential role in cancer invasion/metastasis and chemoresistance. In orthotopic xenograft models of a human bladder cancer cell line, UM-UC-3, metastatic sublines were established from tumors in the liver, lung, and bone. These cells possessed elevated levels of EMT-associated markers, such as Snail, Slug, or CD44, and exhibited enhanced invasion. By microarray analysis, AKR1C1 was found to be up-regulated in metastatic lesions, which was verified in metastatic human bladder cancer specimens. Decreased invasion caused by AKR1C1 knockdown suggests a novel role of AKR1C1 in cancer invasion, which is probably due to the regulation of Rac1, Src, or Akt. An inflammatory cytokine, interleukin-1β, was found to increase AKR1C1 in bladder cancer cell lines. One particular non-steroidal anti-inflammatory drug, flufenamic acid, antagonized AKR1C1 and decreased the cisplatin-resistance and invasion potential of metastatic sublines. These data uncover the crucial role of AKR1C1 in regulating both metastasis and drug resistance; as a result, AKR1C1 should be a potent molecular target in invasive bladder cancer treatment.

Bladder cancer is the seventh most common cancer and ninth leading cause of cancer death in males worldwide^1^. Bladder cancers are clinically divided into two types, non-muscle-invasive bladder cancers (NMIBCs) with 5-year survival rates of 90% and muscle-invasive bladder cancers (MIBCs) with poor prognoses. MIBC frequently exhibits distant metastasis, resulting in 5-year survival rates of less than 6%. As a result, the development of a new therapy to inhibit cancer invasion and metastasis is urgently needed.

The differential molecular machinery involved in NMIBC and MIBC has been established. NMIBCs possess a diploid karyotype and MIBCs exhibit aneuploidy and genomic alterations, such as chromothripsis^2^. In the early stage of NMIBC, FGF receptor 3 mutation and loss of heterozygosity (LOH) for chromosome 9 can frequently be observed^3^, which is followed by additional mutations of PI 3-kinase (PI3K), cyclin D1, or H-Ras^4^. In MIBC, overexpression and mutation of ERBB2 and EGFR have frequently been demonstrated^5^,^6^. The cancer genome atlas (TCGA) analysis revealed four MIBC groups according to mutation and expression profiles that are closely related to tumor suppressors, including p53/Rb, histone modification, SWI/SNF chromatin remodeling, and receptor tyrosine kinases (RTK)/Ras/PI3K such as the FGFR3-TACC3 fusion gene^7^.

The epithelial-mesenchymal transition (EMT) is known to be the initial step of invasion and metastasis in bladder cancer, and this process is related to cancer stemness^8^,^9^. Several transcription factors, such as Snail, Slug, Twist, and ZEB1, are involved in the EMT features that define decreased E-cadherin expression and elevated N-cadherin, fibronectin, and MMP2 levels, resulting in the acquisition of specific mesenchymal morphology and function^10^,^11^. ZEB1 is particularly known as a target of miR200, which is reportedly down-regulated in bladder cancer^12^. The tumor microenvironment, which consists of fibroblasts, endothelial cells, and tumor-associated macrophages, contributes to EMT through producing TGF-β, FGF and IL1-β^13^,^14^.

BCG administration is standard therapy for NMIBCs, and neoadjuvant chemotherapy and/or radiation therapy may be used for MIBCs. The current results of clinical trials suggest that a combination protocol of paclitaxel, radiation and an anti-ERBB2 antibody may be effective for treating localized bladder cancer^15^,^16^. The most common sites of bladder cancer metastasis are the lymph nodes, lungs, liver, and bone^17^; no significant efficacy of molecular targeted therapy has been reported in these metastatic sites^16^. In practice, the treatment options for metastatic bladder cancer are limited to a cisplatin-based combination chemotherapy regimen^18^.

Various metabolic enzymes are involved in the initiation, progression, and prognosis of human cancers^19^. Aldo-keto reductase family 1, member C1 (AKR1C1) is involved in maintaining steroid hormone homeostasis, prostaglandin metabolism, and metabolic activation of polycyclic aromatic hydrocarbons^20^,^21^. Transforming potentials of the AKR1C family in NIH3T3 cells have been reported^22^, and AKR1C2 overexpression is a high-risk factor for bladder cancer^23^. As the AKR1C family is involved in chemotherapy resistance in various cancers, including stomach, colon, lung, and brain cancers^24^,^25^,^26^,^27^, this molecule may play a key role in bladder cancer.

Murine models are potentially useful systems for elucidating the molecular mechanisms underlying the invasion, metastasis, and drug resistance associated with bladder cancer aggressiveness. One of the most useful models involves orthotopic inoculation of bladder cancer cell lines into the mucosal membrane through the urethra^28^,^29^. Using bladder cancer cells that are genetically labeled with luciferase, a single metastatic cell can be monitored *in vivo* and a metastatic subpopulation can be purified^30^. Using this orthotopic xenograft murine model, we investigated the molecular mechanism of bladder cancer metastasis for identifying a therapeutic candidate reagent.

## Results

### Establishment of primary and metastatic cancer cells by orthotopic human bladder cancer xenograft

**Figure 1:**
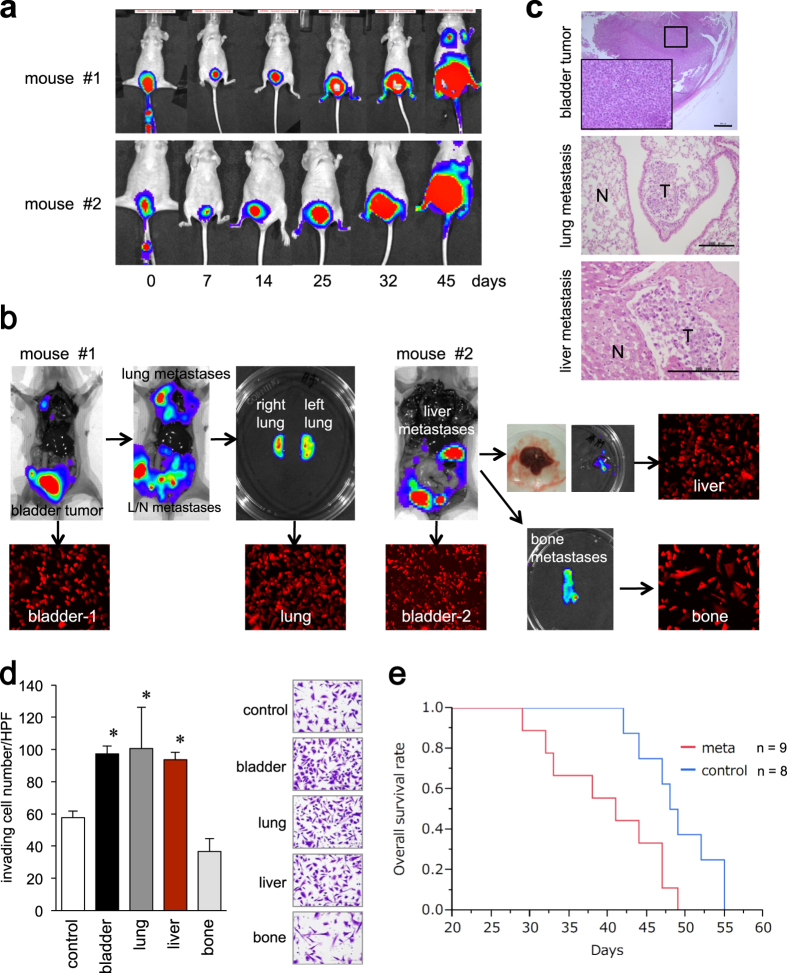
Establishment of UM-UC-3 metastatic sublines in an orthotopic xenograft model. (**a**) UM-UC-3 cells labeled with tdTomato-luc2 were orthotopically implanted into two athymic mice. Tumor growth was monitored using a bioluminescence imaging system on the indicated days. (**b**) After 45 days, the mice were sacrificed with bioluminescent imaging photons that showed the primary bladder tumor and lymph nodes, lung, liver, and bone metastases. Fluorescent microscopy demonstrated tdTomato-expressing tumor cells isolated in the culture dish. (**c**) Excised tumors were fixed and stained with H&E. Primary bladder tumors with a magnified insert (top) as well as lung (middle) and liver (bottom) metastatic tumors. N: normal tissue, T: tumor. (**d**) Matrigel invasion assay. In UM-UC-3, UM-UC-3-tdTomato-luc2 (control), primary bladder tumor cells, and lung, liver, and bone metastatic cells were seeded onto Matrigel-coated transwell chambers. After 24 h of incubation, the invading cells under the filter were counted and depicted as the means ± SD. Representative photographs are shown. *P < 0.05 versus WT. (**e**) Kaplan-Meier curves were constructed to compare survival between the mice implanted with control cells (control: n = 8) versus metastatic cells (meta; lung: n = 5, liver: n = 4). The overall survival of mice implanted with the metastatic cells was significantly worse (*P* < 0.05).

To investigate the molecular mechanisms of bladder cancer metastasis, we established an orthotopic xenograft model using human bladder cancer UM-UC-3 cells that were stably expressing firefly luciferase 2 and tdTomato red fluorescent protein (designated as UM-UC-3-tdTomato-luc2). The cells were inoculated into the bladders of two nude mice through a urethral catheter, and the labeled cancer cells were successfully detected by bioluminescent imaging (BLI) (Fig. 1a). The primary bladder xenograft gradually grew and after 45 days, metastatic tumors could be detected in the lungs, liver, bone, and lymph nodes (Fig. 1a,b), which were confirmed histologically (Fig. 1c). A significant increase in the invasiveness of the UM-UC-3-bladder, UM-UC-3-lung, and UM-UC-3-liver cells was observed compared with the parental cells (control) (Fig. 1d), which is similar to the increased motility according to the chemotaxis assay (Supplementary Fig. 1a). The growth rates of these sublines were approximately equal, except for the UM-UC-3-bone cell line, which displayed slower growth (Supplementary Fig. 1b). Kaplan-Meier analysis of the mice with orthotopically implanted UM-UC-3-lung (n = 5) and UM-UC-3-liver (n = 4) cells demonstrated significantly worse survival than those with the control cells (n = 8, *P* < 0.01; Fig. 1e, Supplementary Fig. 1c), suggesting the metastatic tumors have aggressive features. It should be noted that metastatic bladder cancer models could not be established using the other bladder cancer cell lines J82 and TCC-SUP (Supplementary Fig. 1d).

### AKR1C1 and EMT- and stemness-related genes were increased in metastatic cancer cells

**Figure 2:**
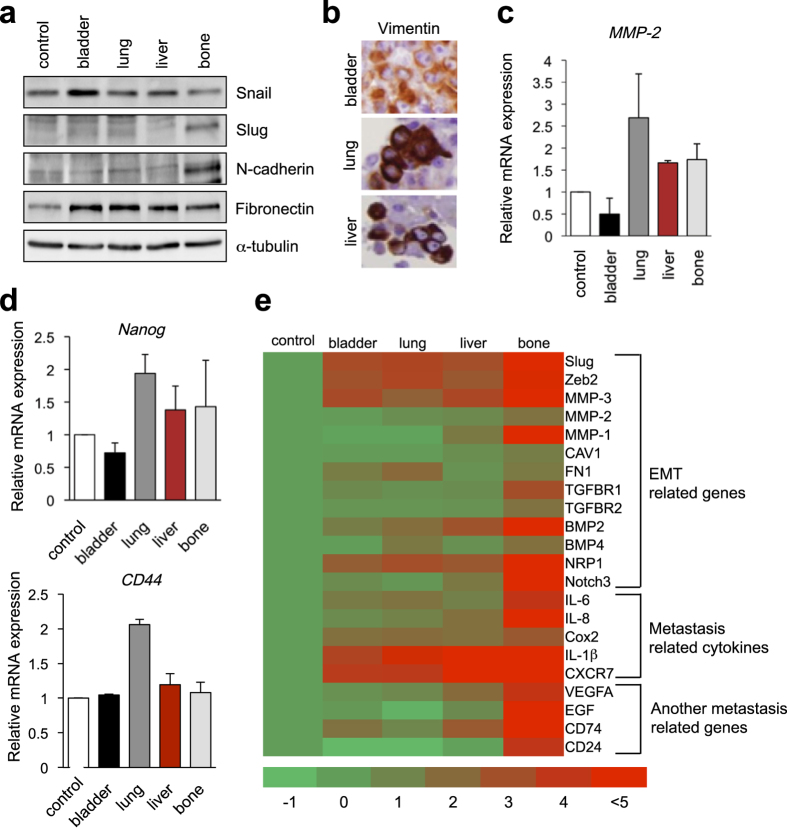
Expression levels of EMT-related molecules in UM-UC-3 sublines, including control, primary bladder, and the metastatic tumor cells in lung, liver, and bone. (**a**) Expression levels of EMT-related proteins were investigated by immunoblotting (IB). α-tubulin was used as a loading control. Cropped images were displayed, and original blots are shown in Supplementary Fig. 9. (**b**) Vimentin expression in murine xenografts was evaluated by immunohistochemistry (IHC). (**c**,**d**) *MMP-2* (**c**) and *Nanog* and *CD44* mRNA expression levels (**d**) were examined by quantitative real-time RT-PCR. (**e**) In microarray analysis, a heat-map of differential gene expression associated with tumor metastasis is displayed. EMT, epithelial-mesenchymal transition.

As invasive potential and aggressiveness were increased in the metastatic cancer cells, we next investigated the role of EMT and the stemness profile in metastatic cell lines. Immunoblot analysis revealed that the UM-UC-3-bladder cells had higher expression levels of Snail and fibronectin, EMT markers, compared with the control cells (Fig. 2a). An increase in the fibronectin expression level was also observed in all metastatic sublines, whereas the UM-UC-3-bone cells had additional enhancement of Slug and N-cadherin (Fig. 2a). To verify EMT, the vimentin expression levels were increased in lung and liver micrometastatic tumor cells compared to primary bladder cancer according to immunohistochemistry (Fig. 2b). The expression levels of *MMP-2* and stemness markers, such as *Nanog* and *CD44*, were also increased in the three metastatic cell lines (Fig. 2c,d). To assess their stemness features, decreased mRNA levels of *cytokeratin CK18* and increased mRNA levels of *p63* were observed by microarray analysis (data not shown).

**Figure 3:**
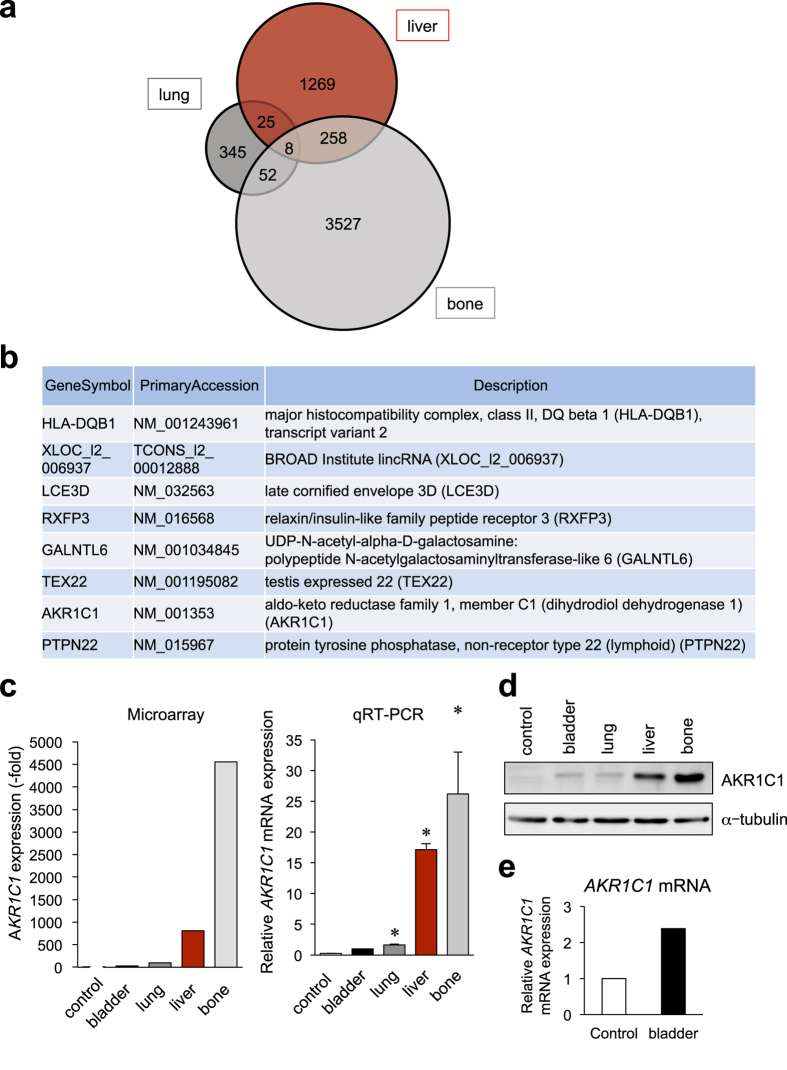
*AKR1C1* was commonly upregulated in metastatic UM-UC-3 sublines. (**a**) The Venn diagram displays the numbers of genes upregulated more than 2-fold among the 3 metastatic sublines (lung, liver, and bone) compared with the primary bladder tumor cells (bladder-1 vs. lung, bladder-2 vs. liver or bone). (**b**) Eight genes commonly up-regulated in the three metastatic sublines compared to the bladder subline. (**c**) *AKR1C1* mRNA expression levels among UM-UC-3 sublines were examined by microarray (left) and real-time RT-PCR (right). *P < 0.05 vs. bladder. (**d**) AKR1C1 protein levels were investigated by immunoblotting. Cropped images are displayed, and original blots are shown in Supplementary Fig. 10. (**e**) The *AKR1C1* mRNA expression levels were examined between UM-UC-3-tdTomato-luc2 (control) and the primary bladder cancer-derived cells in an orthotopic murine xenograft.

To identify the molecules responsible for bladder cancer metastasis, gene expression profiles were investigated by microarray analysis using the control UM-UC-3 line, the primary UM-UC-3-bladder line, and three metastatic UM-UC-3 sublines. Increased expression levels of EMT-related molecules, such as *Slug*, *Zeb2*, *MMP-3*, *MMP-1*, *TGFBR1*, *BMP2*, and *NRP1*; metastasis-related cytokines, such as *IL-6*, *IL-8*, *Cox2*, *IL-1*β, and *CXCR7*; and other metastasis-related genes, such as *VEGFA*, *EGF*, *CD74*, and *CD24*, were observed in the primary and metastatic tumors (Fig. 2e). We focused on the genes that are commonly up-regulated more than two-fold in all three metastatic tumor cells compared with the primary bladder tumor-derived cells (Fig. 3a), and eight genes were identified (Fig. 3b). Among them, increased mRNA expression of *aldo-keto reductase family 1*, *member C1* (*AKR1C1*) was confirmed in metastatic sublines by qRT-PCR (Fig. 3c), whereas the levels of other genes were not significantly elevated (Supplementary Fig. 2a,b). The expression levels of the AKR1C1 protein were also elevated in the metastatic tumors, particularly in UM-UC-3-liver and UM-UC-3-bone cells (Fig. 3d). The *AKR1C1* expression level in primary bladder tumors was elevated 2.4-fold compared to that in control cells (Fig. 3e).

### IL-1β induces *AKR1C1* expression and elimination of AKR1C1 decreases UM-UC-3 cell invasion

**Figure 4:**
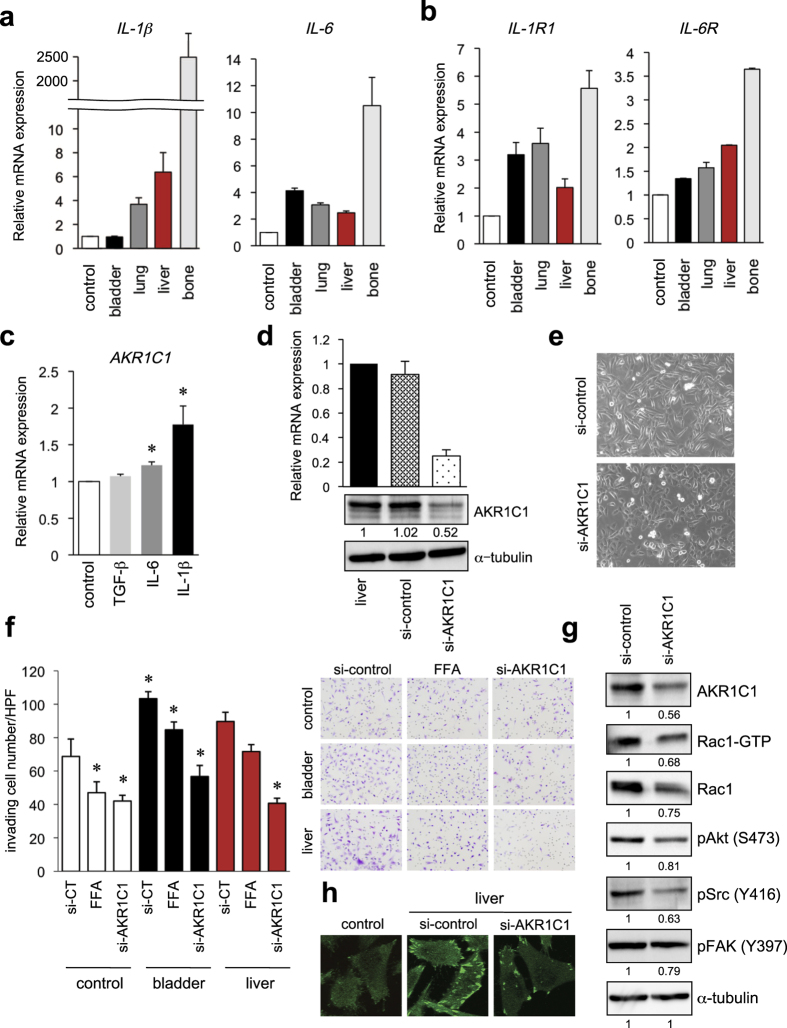
IL-1β- and IL-6-induced *AKR1C1* expression in the bladder cancer cell line and *AKR1C1* knockdown suppresses the invasion of UM-UC-3 cells *via* a decline in the Src and FAK phosphorylation as well as Rac1 activity. (**a**,**b**) *IL-1*β and *IL-6* (**a**) and *IL-1R1* and *IL-6R* (**b**) expression levels in UM-UC-3 sublines were examined by real-time RT-PCR. (**c**) UM-UC-3-control cells were treated with TGF-β1 (5 ng/mL), IL-6 (10 ng/mL), and IL-1β (20 ng/mL). *AKR1C1* mRNA expression levels were examined by real-time qRT-PCR. *P < 0.05 vs. WT. (**d**,**e**) The liver-metastatic UM-UC-3 cells were transfected with siRNA targeting *AKR1C1* or its control. (**d**) *AKR1C1* mRNA and protein expression levels were examined by real-time qRT-PCR after 48 h (upper) and IB after 72 h (lower). (**e**) Photomicrographs of UM-UC-3 cells as indicated were taken under bright-field illumination at 72 h after transfection. (**f**) Effect of AKR1C inhibition in a Matrigel invasion assay. UM-UC-3-control, primary bladder cells, and liver metastatic cells were treated with FFA or siRNA targeting *AKR1C1*, which was followed by a Matrigel invasion assay. *P < 0.05 vs. si-control (CT). (**g**) UM-UC-3-liver metastatic cells were transfected with si-*AKR1C1*; after 72 h, the lysate was subjected to IB. Cropped images were displayed, and original blots were shown in Supplementary Fig. 11. (**h**) UM-UC-3-control and UM-UC-3-liver metastatic cells were transfected with si-*AKR1C1*; after 72 h, immunofluorescence of paxillin was performed.

Next, the molecular mechanisms underlying the increased *AKR1C1* expression levels were analyzed. We focused on IL-1β, IL-6, and TGF-β1 because these are well-known cancer progression-related factors for many malignancy types, including bladder cancer^14^. The qRT-PCR analyses confirmed that the *IL-1* and *IL-6* expression levels and those of their corresponding receptors were increased in metastatic tumors (Fig. 4a,b). To assess a potential role for these factors in *AKR1C1* expression, control UM-UC-3 cells were treated with IL-6 (10 ng/mL), IL-1β (20 ng/mL), or TGF-β1 (5 ng/mL), and qRT-PCR was used to confirm the up-regulation of *AKR1C1* mRNA in response to IL-6 and IL-1β stimulation (Fig. 4c). For verification, the cytokine effects on other bladder cancer cell lines, such as TCC-SUP and 5637, were examined, and IL-1β increased the *AKR1C1* levels in both cell lines (Supplementary Fig. 3).

To examine the role for *AKR1C1* in bladder cancer metastasis, we eliminated *AKR1C1* in the UM-UC-3 cells using a siRNA technique. The knockdown efficiency of *AKR1C1* was validated by both the mRNA and protein levels (Fig. 4d). UM-UC-3-liver cells had a spindle shape, suggesting mesenchymal features, whereas the *AKR1C1* knockdown cells had a flat, cobblestone-like morphology (Fig. 4e). A Matrigel invasion assay demonstrated that the numbers of invading cells were significantly reduced by *AKR1C1* knockdown in the control UM-UC-3, UM-UC-3-bladder, and UM-UC-3-liver cells (Fig. 4f). As the enzymatic activity of *AKR1C3* has been shown to be suppressed by non-steroidal anti-inflammatory drugs (NSAIDs)^31^, we tested the effect of one NSAID, flufenamic acid (FFA), and found that the invasion potential was significantly suppressed in the control and metastatic UM-UC-3 cells (Fig. 4f). Similar results were obtained using another siRNA for AKR1C1 (si-AKR1C1 #2, Supplementary Fig. 4a**–c**). Notably, siRNA-based *AKR1C1* depletion remarkably diminished the expression of Rac1 and phosphorylation levels of Akt (S473), Src (Y416), and FAK (Y397) in the metastatic UM-UC-3-liver cells (Fig. 4g) in addition to numbers of focal adhesion like paxillin (Fig. 4h, Supplementary Fig. 4d), which might indicate a possible role for AKR1C1 in regulating actin cytoskeleton and forming focal adhesion contacts in bladder cancer *via* Src/FAK/Rac1 complexes.

### Inhibition of AKR1C activity reversed cisplatin resistance in metastatic bladder cancer cells

**Figure 5:**
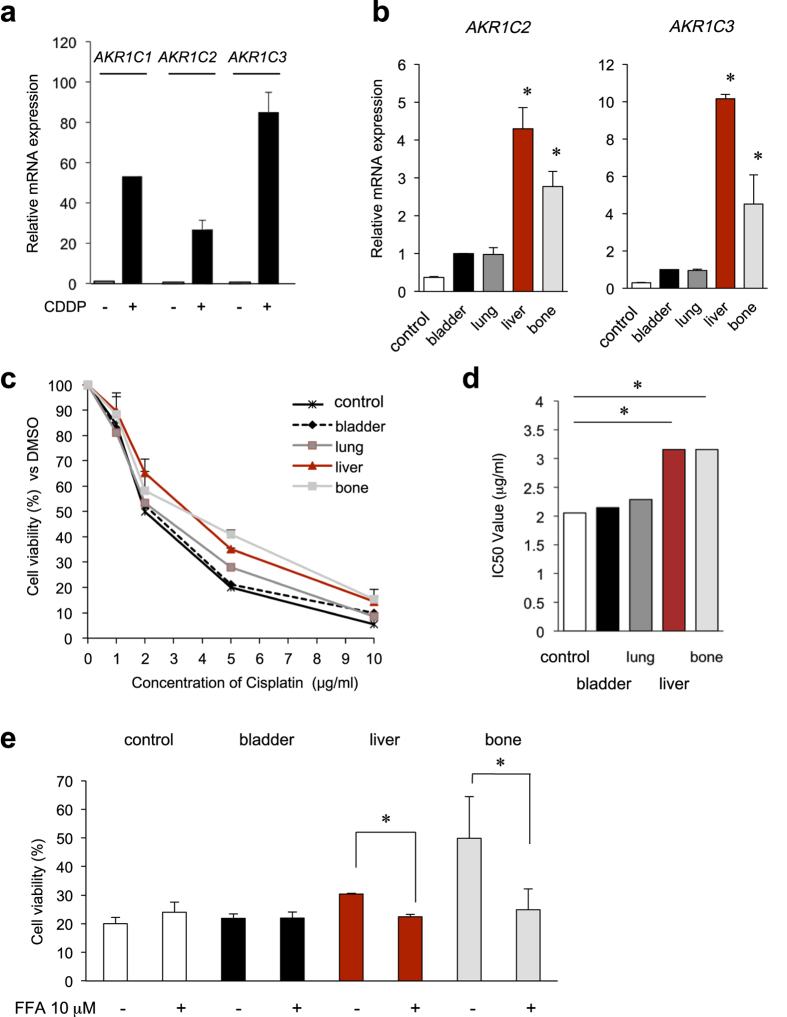
Inhibition of AKR1C activities reverses cisplatin resistance in UM-UC-3-liver- and bone metastatic cells. (**a**) *AKR1C1*, *AKR1C2*, and *AKR1C3* mRNA expression levels in UM-UC-3 cells treated with or without 1 μM cisplatin for 1 month were examined by real-time RT-PCR. (**b**) *AKR1C2* and *AKR1C3* mRNA expression levels among UM-UC-3 sublines were examined by real-time RT-PCR. (**c**,**d**) Sensitivity of UM-UC-3 sublines to cisplatin. UM-UC-3 sublines were treated with cisplatin at the indicated concentrations for 3 days, and cell viability was calculated as a percentage with respect to the control (DMSO). (**d**) The IC_50_ value is displayed on the graph. *P < 0.05 vs. WT. (**e**) Effects of AKR1C inhibitor FFA on cisplatin resistance in UM-CU-3 metastatic cells. UM-UC-3-control, primary bladder, and liver and bone metastatic cells were treated with 5 μM cisplatin for 72 h combined with either 10 μM FFA or DMSO. *P < 0.05 vs. DMSO.

Overexpression of AKR1C isoforms, AKR1C1, AKR1C2, and AKR1C3, is associated with drug resistance in patients with various cancers, including stomach, lung, ovary, and colon cancer^16^,^17^,^18^,^19^,^20^. Therefore, it was of interest to evaluate the role of AKR1C isoforms in the chemotherapeutic response of cisplatin in bladder cancer cells. In the UM-UC-3, 5637, and J82 cell lines, long-term treatment with cisplatin significantly increased the expression levels of all *AKR1C* isoforms (Fig. 5a and Supplementary Fig. 5a,b), although the basal AKR1C1 expression levels were different among these cells (Supplementary Fig. 5c). For the orthotopic xenograft model, the metastatic tumor cells, particularly the liver and bone cells, possessed significantly higher expression levels of *AKR1C1*, *AKR1C2* and *AKR1C3* (Figs 3c and 5b), corresponding with increased cisplatin resistance (Fig. 5c,d). Furthermore, the pharmacological AKR1C inhibitor, FFA, significantly restored the sensitivity of the metastatic tumor cells (liver and bone) to cisplatin (Fig. 5e), suggesting a requirement of AKR1C enzymatic activity for cisplatin resistance. These results illustrate that AKR1C1 confers chemoresistance to cisplatin, and FFA can suppress AKR1C1-dependent resistance in metastatic cells, resulting in increased sensitivity to chemotherapy.

### Enhancement of AKR1C1 expression in metastatic human bladder cancer lesions

**Figure 6:**
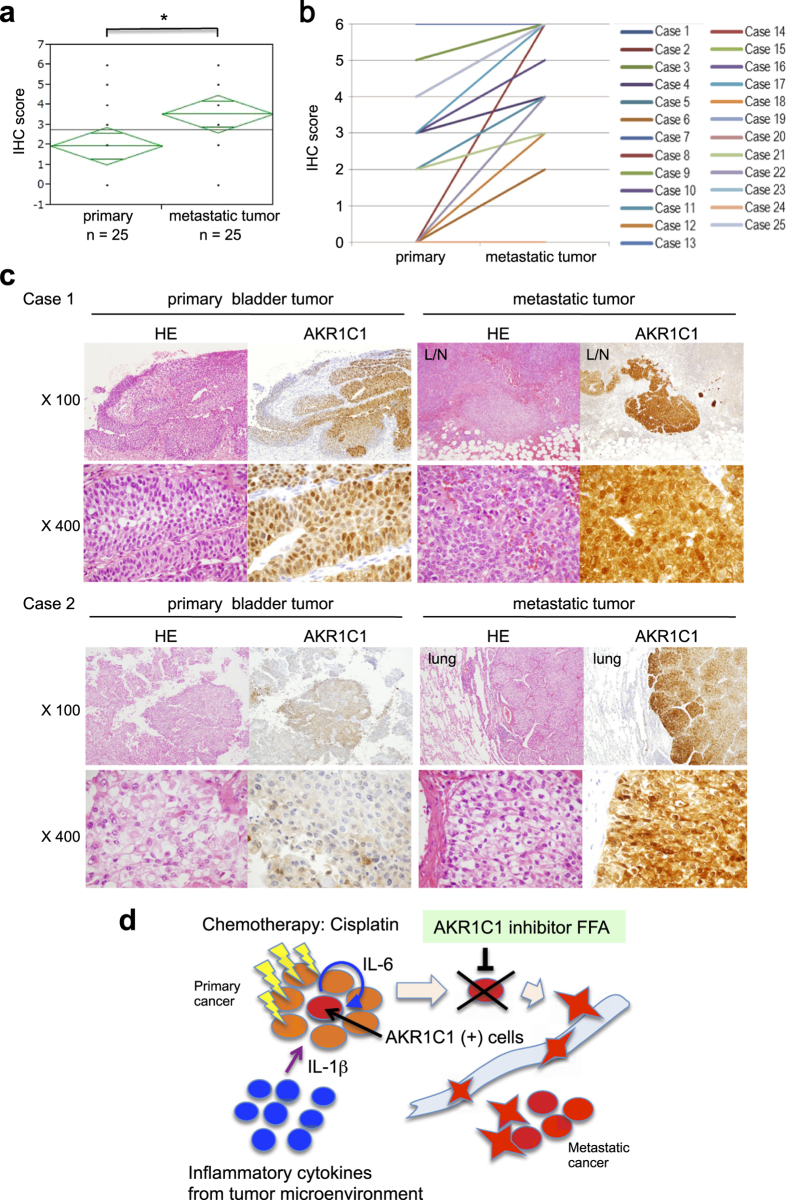
Increased AKR1C1 expression in human metastatic tumors. AKR1C1 expression levels in human bladder cancer specimens were examined by IHC. The total score for AKR1C1 staining (**a**) and the matched-pair plot of 25 cases (**b**) are presented. Primary tumor (n = 25) and metastatic tumor (n = 25). *P < 0.05 vs. primary. (**c**) Primary bladder tumor and metastatic specimens to the lymph nodes or lungs were subjected to H&E staining and AKR1C1 immunohistochemistry. (**d**) The putative mechanism of AKR1C1-dependent bladder cancer acquisition of therapy-resistance, invasion, and metastatic potential.

To ensure clinical significance, we examined the *AKR1C1* levels using surgically resected specimens derived from bladder cancer patients, including 33 primary and 5 metastatic lesions involving the lymph nodes (n = 3), lungs (n = 1), and liver (n = 1). RT-PCR analysis demonstrated that the metastatic tumors exhibited higher levels of *AKR1C1* mRNA compared with the primary sites (*P* = 0.008; Supplementary Fig. 6). For verification, immunohistochemical analysis was performed using 25 matched-paired samples of primary and metastatic human bladder cancer lesions (Supplementary Table 2). The AKR1C1 expression level was evaluated as the sum of the intensity score (0–3) and the proportion score (0–3) for AKR1C1 immunostaining (Supplementary Fig. 7a,b). In 17 out of 25 cases, higher AKR1C1 expression was observed in the metastatic lesions affecting the lymph nodes, lung, and liver compared with the paired primary tumors (Fig. 6a–c and Supplementary Fig. 7c).

## Discussion

To advance human bladder cancer treatment, it is necessary to understand the molecular mechanisms underlying tumor invasion, metastasis, and drug resistance. In this study, we evaluated orthotopic mouse xenograft models in which cancer cells were inoculated through the urethra. The cells spontaneously attached to the bladder mucosa surface and formed solid tumors, resulting in natural tumor growth and metastasis to other organs. We utilized the bladder cancer cell line, UM-UC-3, possessing a typical MIBC gene profile with LOH of chromosome 9, p53 mutation, and ERBB2 overexpression without FGFR3 mutation^32^,^33^. In our model, circulating cancer cells were observed in the bloodstream of the mice^34^, and we could establish three metastatic sublines from the nodules found in the lungs, liver, and bone.

In these three cell lines, EMT markers, including *Snail*, *Slug*, *ZEB2*, *N-cadherin*, and vimentin, were elevated and had increased *in vitro* invasion potential (Figs 1d and 2a,b,e). Injected bladder cancer cells grown in the mucosa also exhibited enhanced invasiveness with their EMT profiles compared to wild type UC-UM-3 cells. Cancer stem cells also play a major role in tumor recurrence and metastasis in various cancers, including bladder cancer^35^,^36^. In our xenograft model, *CD44* and *Nanog*, as stemness markers, were significantly up-regulated in the lung metastatic subline compared with the control cells (Fig. 2d). As *ERBB2* is frequently overexpressed in metastatic lesions^37^, mild enhancement of *ERBB2* was observed in all three metastatic cell lines by microarray analysis (data not shown).

The UM-UC-3-lung and UM-UC-3-liver metastatic sublines exhibited aggressive invasion features (Fig. 1d) and *in vivo* tumor formation (data not shown). Kaplan-Meier analysis of the mice implanted with metastatic sublines (lung: n = 5, liver: n = 4) demonstrated significantly worse survival than those implanted with the control cells (n = 8, *P* < 0.01; Fig. 1e). Additionally, the UM-UC-3-bone metastatic cells demonstrated less invasiveness in the Matrigel invasion assay (Fig. 1d) and lower growth potential *in vitro* (Supplementary Fig. 1b) and *in vivo* (data not shown), although the cells exhibited higher expression levels of EMT markers and metastasis-associated genes (Fig. 2e). This discrepancy between the expression profile and biological phenotype of the UM-UC-3-bone cells can be from the microenvironment-dependent growth of the cells. UM-UC-3-bone cells can grow inside the surrounding physically hard materials, including calcium phosphate in bone; as a result, they may not properly move in conventional *in vitro* assays. Further investigation is required to delineate the precise characteristics of metastatic bone cells.

In our metastatic model system, *AKR1C1* was identified as one of the commonly up-regulated genes in all three metastatic cell lines. In this study, immunohistochemistry demonstrated that AKR1C1 was highly expressed in metastatic lesions of human bladder cancer patients (Fig. 6a–c and Supplementary Fig. 7c). Although a higher AKR1C3 level was reported in the metastatic bone tumors of the prostate cancer^38^, the molecular mechanism of AKR1C1-dependent bladder cancer metastasis had not been previously described.

We investigated the mechanism for the increased AKR1C1 levels in the bladder cancer cell lines and examined whether pro-inflammatory mediators and growth factors, which are related to tumor progression, regulate *AKR1C1* expression in bladder cancer cells^26^,^39^. Our microarray and qRT-PCR data suggested that metastatic tumor cells possess higher expression levels of endogenous *IL-6* and *IL-1*β and their receptors (Figs 2e and 4a,b). IL-1β enhanced the expression of *AKR1C1* in the three bladder cancer cell lines, UM-UC-3, TCC-SUP, and 5637 cells (Fig. 4c and Supplementary Fig. 3). The significance of this *IL-1*β dependent increase in *AKR1C1* was confirmed in clinical specimens of human bladder cancer tissues (Supplementary Fig. 8).

The molecular machinery for the AKR1C1-dependent up-regulation of EMT and invasiveness was examined with *AKR1C1* knockdown bladder cancer cells that exhibited less *in vitro* invasiveness (Fig. 4f). Steroid hormones may affect cancer cell migration; for example, 17β-estradiol suppresses IL-6-dependent tyrosine phosphorylation of Src, p130^Cas^, and paxillin in gastric cancer cells^40^. As a result, inhibition of 17β-estradiol by AKR1C1 may recover cell motility in cancer cells. In fact, for the *AKR1C1* knockdown cells, Src and FAK phosphorylation was decreased (Fig. 4g). In addition, AKR1C family proteins are implicated in the metabolism of small GTPases, such as Ras, Rho, and Rac, through regulating prenylation^41^. AKR1C may produce geranylgeranyl pyrophosphate as the substrate of geranyl-geranyl transferase, providing higher levels of mature small GTPases, such as Rac^41^. The decreased concentration of Rac1 in *AKR1C1* knockdown cells (Fig. 4g) may reflect the involvement of this pathway.

Recently, AKR1C2 was reported to regulate cisplatin resistance in bladder cancer^42^. The established liver and bone metastatic cells in this study exhibited higher *AKR1C1*, *AKR1C2* and *AKR1C3* expression levels (Figs 3c,d and 5b), and this overexpression appeared to correlate with cisplatin resistance (Fig. 5c,d). It is speculated that in cisplatin-resistant cells, AKR1C may reduce the levels of cytotoxic lipid peroxidative products from an aldehyde, such as 4-hydroxy-trans-2-nonenal (HNE) and 4-oxo-2-nonenal (ONE)^24^. To reverse this resistance, we examined the effect of a chemical inhibitor of the AKR1C family, flufenamic acid (FFA), and found that FFA could reverse the cisplatin resistance of metastatic liver and bone UM-UC-3 cells (Fig. 5e). Furthermore, AKR1C1 expression was associated with cancer stem cells in lung and thyroid cancer^43^,^44^. Therefore, AKR1C1 contributes to the development of chemoresistance and induction of stemness-like features in metastatic bladder cancer.

In primary localized bladder cancer, particularly after cisplatin exposure, the surrounding tumor microenvironment may promote IL-1β production by inflammatory lymphocytes. These inflammatory microenvironments with IL-1β production may up-regulate the *AKR1C1* levels in a specific cell clone, resulting in cisplatin-resistance and increased invasiveness, which allows the cells to penetrate into blood vessels and circulate throughout the entire body. Finally, these cells can attach to organ tissues, such as the lungs, liver, and bone, to form metastatic nodules. A limitation of this study is the use of a single cell line, UM-UC-3, due to lack of tumor forming potentials of other cells, including T24, J82, and TCC-SUP. As FFA suppressed cisplatin resistance and cell invasion *in vitro*, FFA may have clinical significance in reversing drug resistance and suppressing metastasis, improving the prognosis of bladder cancer patients (Fig. 6d).

## Materials and Methods

### Cell lines, reagents, and antibodies

The human bladder cancer cell line, UM-UC-3, was purchased from the American Type Culture Collection (Manassas, VA). Wild-type UM-UC-3 and its sublines were established in orthotopic xenograft models (primary bladder cancer, and lung, liver, and bone metastatic cells) cultured in Dulbecco’s modified Eagle’s medium (DMEM) containing 10% fetal bovine serum (FBS, Gibco^®^-life Technologies, Grand Island, NY, USA). Flufenamic acid and cisplatin were purchased from Sigma-Aldrich (St. Louis, MO). Human recombinant interleukin (IL)-6 and transforming growth factor-β1 (TGF-β1) were obtained from R&D Systems (Minneapolis, MN), and human recombinant IL-1β was purchased from PeproTech (Rocky Hill, NJ). Antibodies were purchased from the following suppliers: the antibody to aldo-keto reductase 1C1 (AKR1C1) was from Lifespan Biosciences (Seattle, WA); those for Snail, Slug, N-cadherin, phospho-FAK (Y397), phospho-Akt (S473), and phospho-Src (Y416) were from Cell Signaling Technology (Beverly, MA); those for fibronectin and Rac1 were from BD Transduction; and that for α-tubulin was purchased from Sigma-Aldrich.

### Establishment of UM-UC-3 cells stably expressing tdTomato-Luc2

UM-UC-3 cells were stably transfected with pCSII-CMV-tdTomato-Luc2 (kindly provided by Dr. Kyoko Hida, Hokkaido University, Sapporo), which was followed by selection using 0.5 mg/mL bleomycin. The resulting live cells were sorted by fluorescence-activated cell sorting (FACS; Aria II, BD, Japan), and the cells with excessive tdTomato expression were isolated. Luciferase activity was examined with a luciferase assay (Promega, Madison, WI).

### Orthotopic xenograft model

We have previously reported on the methods for generating this murine model^28^. Six- to eight-week-old female nude mice, Balb/cA Jcl nu/nu (Clea Japan, Inc., Tokyo, Japan), were used for an orthotopic xenograft model. After the mice were anesthetized with either 1.75% isoflurane or intraperitoneal sodium pentobarbital (60 mg/kg), the bladder was pretreated with 100 μL of 0.1 mg/mL poly-*L*-lysine (Sigma) for 20 min through the urethra with vascular catheters (24G; Terumo, Tokyo, Japan). After that, the bladder was flushed with 100 μL of phosphate-buffered saline (PBS), and 5 × 10^6 ^UM-UC-3-tdTomato-luc2 cells in 50 μL of PBS were inoculated into the bladder. For the 3 h before catheter removal, mice were placed under anesthesia. Bioluminescent imaging (BLI) was performed with the IVIS Spectrum imaging system (Caliper Life-Sciences, Hopkinton, MA) using a post-intraperitoneal injection of VivoGlo Luciferin *In Vivo* Grade (Promega). Xenograft-derived cells were collected from the primary site, and three metastatic lesions from the lungs, liver, and bone were used to establish the following sublines: UM-UC-3-bladder-1 and UM-UC-3-lung from mouse 1 and UM-UC-3-bladder-2, UM-UC-3-liver, and UM-UC-3-bone from mouse 2. All animal experiments were conducted in accordance with the guidelines of Hokkaido University Manual for Implementing Animal Experimentation and approved by Institutional Animal Care and Use Committee at Hokkaido University Graduate School of Medicine (Number 12-0092). All researchers who performed procedures using live animal were pre-approved by the Animal Welfare Committee of Hokkaido University based on their completion of required animal use and care training and acceptable previous experience with animal experiments. All ethical issues related to animal experiments and human pathological specimens were discussed and approved by the Ethics Committee of Hokkaido University Graduate School of Medicine.

### Establishment of primary and metastatic tumor cells in an orthotopic xenograft model

At 45 days post-inoculation with UM-UC-3 cells, the primary and metastatic tumors were identified with the IVIS imaging system. The mice were euthanized, and tumor tissues were excised and incubated in medium with collagenase for 30 min at 37 °C. After filtration, the cells were cultured with bleomycin to select tumor cells for two weeks, and the primary and metastatic tumor cells were established (Mouse #1; bladder-1 and lung metastatic tumors; Mouse #2; bladder-2, liver- and bone metastatic tumors). The established tumor cells were verified using a fluorescence microscope to detect tdTomato^45^.

### RNA extraction and quantitative real-time RT-PCR

Total RNA isolation and first-strand cDNA synthesis were performed as previously described^46^. The primer sequences are listed in Supplementary Table S1. Quantitative real-time polymerase chain reaction (qRT-PCR) was performed using the StepOne real-time PCR system (Applied Biosystems, Foster City, CA). Data were normalized to the *glyceraldehyde 3-phosphate dehydrogenase* (*GAPDH*) expression level and are expressed as the fold change relative to control.

### Microarray analysis

Total RNA was extracted from parental UM-UC-3 and its sublines derived from orthotopic xenografts (bladder-1, bladder-2, lung, liver, and bone), and microarray analysis was performed using SurePrint G3 Human Gene Expression 8 × 60K v2 (Agilent Array^®^). Gene expression was analyzed in bladder-1 *versus* lung and bladder-2 *versus* liver and bone cell lines.

### Cell viability assay

Cells (1 × 10^5^) suspended in complete DMEM were plated in 60-mm dishes. After 24 h, cisplatin and FFA were added to the culture medium. Three days after treatment, the cells were trypsinized and counted.

### Invasion assay

The invasion assay was performed as previously described^47^. Briefly, 1 × 10^5^ cells suspended in serum-free medium were seeded onto the upper chamber. Medium containing 10% FBS as a chemoattractant was added to the lower chamber. After 22 h, invaded cells were counted at 200x magnification in at least 3 randomly selected fields.

### Immunoblotting

Cells were washed twice with cold PBS and lysed in a buffer containing 0.5% NP-40, 10 mM Tris-HCl (pH 7.4), 150 mM NaCl, 1 mM EDTA, 50 mM NaF, 1 mM PMSF, and 1 mM Na_3_VO_4_, and the lysate was clarified by centrifugation at 15,000 rpm for 10 min. The supernatants were then subjected to 8–12% SDS-PAGE. Separated proteins were transferred to polyvinylidene difluoride membranes and blocked using Tris-buffered saline containing Tween-20 (TBS-T) with 5% skim milk at room temperature for 1 h. Primary antibodies were incubated at 4 °C overnight and then washed with TBS-T, which was followed by incubation with secondary antibodies. The membranes were washed 3 times in TBS-T, and the signal was developed using ECL (GE Healthcare, Little Chalfont, UK), which was followed by detection using the LAS4000 (GE Health Care).

### Knockdown of AKR1C1

Small interfering RNAs (siRNAs) targeting human *AKR1C1* (si-*AKR1C1 #1*: AATTGTTCTGGTTGCCTATAG, si-*AKR1C1 #2*: TTGGCCAGAAAGGAAAGACAA) were purchased from Qiagen (Valencia, CA). Cells (5 × 10^6^) were transfected with 50 nM of si-*AKR1C1* or si-scramble as control using HiPerfect transfection reagent (Qiagen), and they were subjected to real-time RT-PCR and immunoblotting after 48 and 72 h, respectively.

### Immunofluorescence of paxillin

Immunofluorescence of paxillin was performed according to the previous report^47^. Briefly, cells were fixed with 3% paraformaldehyde for 15 min at room temperature, permeabilised with 0.1% Triton X-100 in PBS for 4 min at room temperature, and then incubated with 1% bovine serum albumin to block nonspecific binding of antibodies. The cells were further incubated with anti-paxillin antibody (1:1000 dilution) overnight at 4 °C, after which immune complexes were detected by incubation for 1 h at room temperature in the dark with AlexaFluor488-conjugated secondary antibodies (1:250 dilution). Images were acquired with an FV-10i confocal microscope (Olympus, Tokyo, Japan).

### Immunohistochemical analysis

Formalin-fixed paraffin-embedded tissues, including human bladder cancer specimens (surgically resected in Department of Urology in Hokkaido University Hospital, Sapporo, Japan) that were obtained with informed consent, were sectioned and stained with hematoxylin and eosin (H&E). Immunohistochemistry (IHC) was performed using an antibody to AKR1C1 (Lifespan Biosciences). All ethical issues related to the animal experiments and human pathological specimens were discussed and approved by the Ethics Committee of Hokkaido University Graduate School of Medicine and Hokkaido University Hospital, respectively. Experiments were performed in accordance with the relevant Ethical Guidelines.

### Statistical analysis

Data are presented as the mean and standard deviations (S.D.) for at least 3 independent experiments. Student’s *t*-test, Mann-Whitney U-test, or Spearman’s test were used to analyze significant differences. JMP^®^ version 10 (SAS Institute, Inc., Cary, NC) was used for all calculations. *P* < 0.05 was considered statistically significant.

## Additional Information

**How to cite this article**: Matsumoto, R. *et al*. Aldo-keto reductase 1C1 induced by interleukin-1β mediates the invasive potential and drug resistance of metastatic bladder cancer cells. *Sci. Rep*. **6**, 34625; doi: 10.1038/srep34625 (2016).

## Supplementary Material

Supplementary Information
