# Supplementary material for: Aldo-keto reductase 1C1 induced by interleukin-1β mediates the invasive potential and drug resistance of metastatic bladder cancer cells
Source: Sci Rep. 2016 Oct 4;6:34625. doi: 10.1038/srep34625 (PMC5048132; doi:10.1038/srep34625)
Supplement: Supplementary Information [file srep34625-s1.pdf]

Supplementary information

Aldo-keto reductase 1C1 induced by interleukin-1β mediates the invasive potential and drug resistance of metastatic bladder cancer cells

Ryuji Matsumoto[1,2], Masumi Tsuda[1], Kazuhiko Yoshida[1,3], Mishie Tanino[1], Taichi Kimura[4], Hiroshi Nishihara[4], Takashige Abe[2], Nobuo Shinohara[2], Katsuya Nonomura[2], and Shinya Tanaka[1,4]

Authors' Affiliations: [1]Department of Cancer Pathology, Hokkaido University Graduate School of Medicine, N15, W7, Kita-ku, Sapporo 060-8638, Japan. [2]Department of Renal and Genitourinary Surgery, Hokkaido University Graduate School of Medicine, N15, W7, Kita-ku, Sapporo 060-8638, Japan. [3]Department of Urology, Tokyo Women's University Hospital, Shinjuku-ku, Japan. [4]Department of Translational Pathology, Hokkaido University Graduate School of Medicine, N15, W7, Kita-ku, Sapporo 060-8638, Japan.

a

b

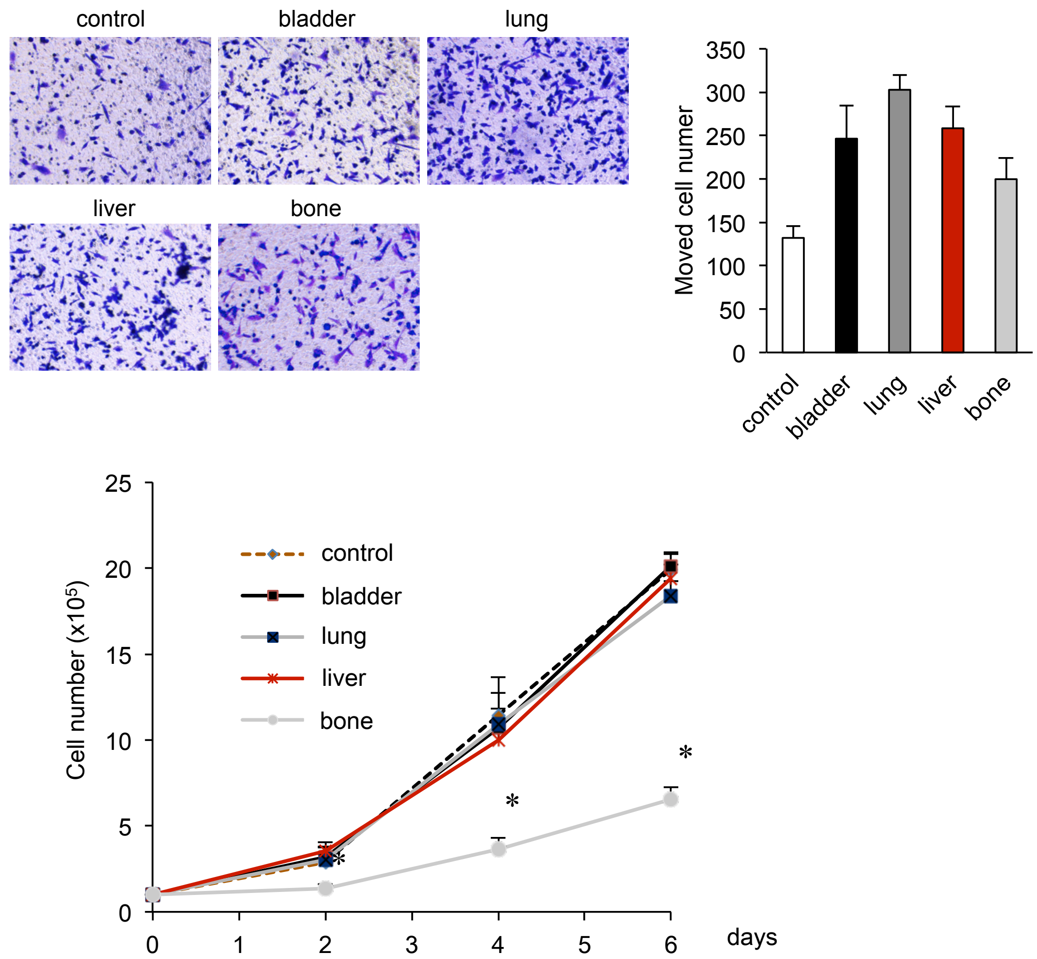

Supplementary Figure 1. (**a**) 1×10[5] cells of control, primary bladder cancer-derived, and three metastatic UM-UC3 cells were subjected to chemotaxis assay. (**b**) *In vitro* cell growth assay. 1 × 10[5] UM-UC-3-WT (wild-type), primary bladder tumor cells, and lung-, liver-, and bone-metastatic tumor cells were cultured in DMEM containing 10% FBS. The cells were counted under a microscope on days 2, 4, and 6. **P* < 0.05 vs. WT. (**c**) In Fig. 1e, 5 × 10[6] UM-UC-3 cells (control: n = 8, metastatic tumor cells: n = 9 (liver: n = 4, lung: n = 5)) were orthotopically inoculated into the bladder of nude mice, and bioluminescent imaging was performed using IVIS Spectrum imaging system after 7, 14, 21, and 28. (**d**) 3 × 10[6] J82 and 8 × 10[6] TCC-SUP bladder cancer cells were orthotopically inoculated into the bladder of nude mice, and bioluminescent imaging was performed using IVIS Spectrum imaging system post-intraperitoneal injection of VivoGlo Luciferin.

Supplementary Figure 1

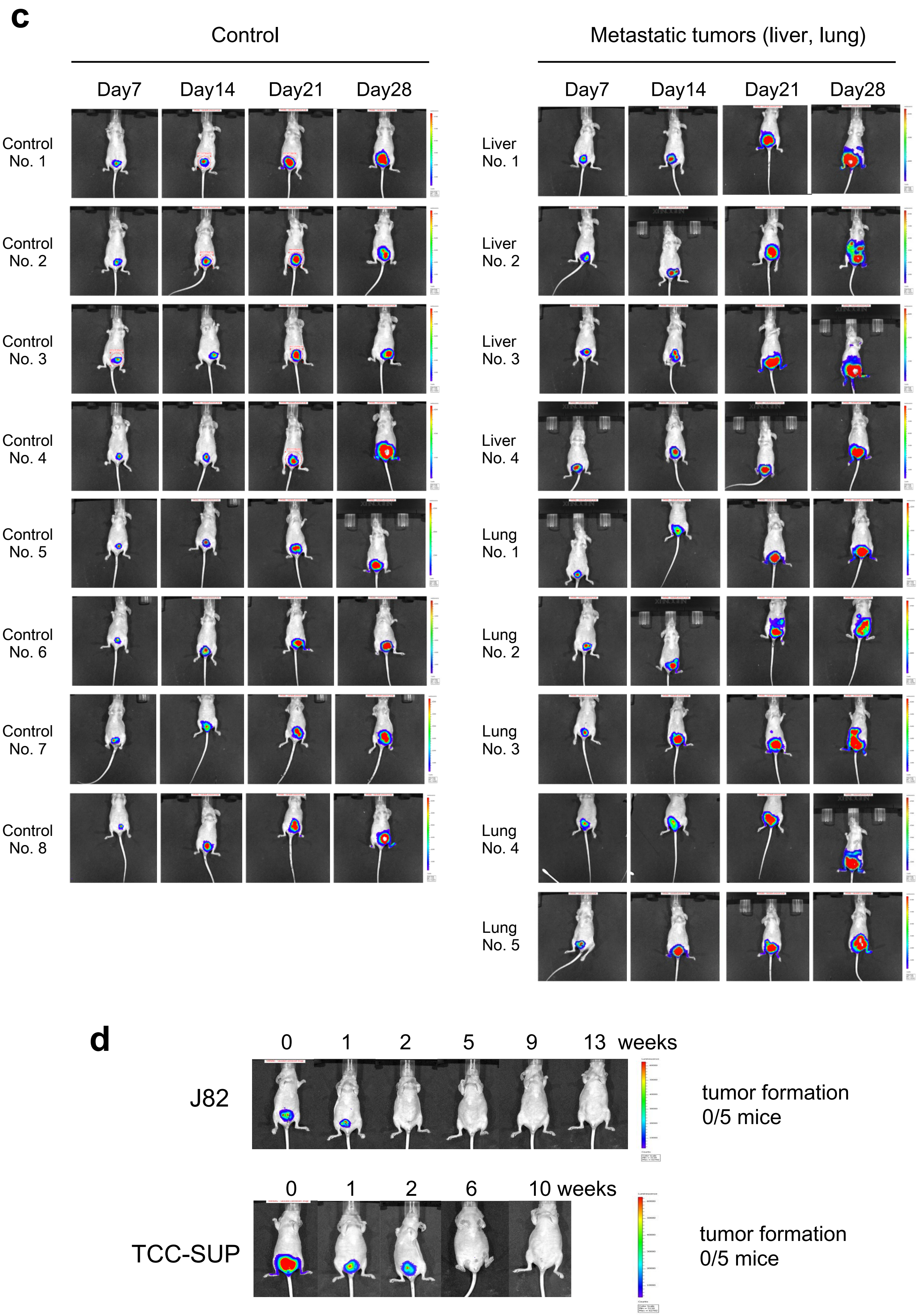

Supplementary Figure 1

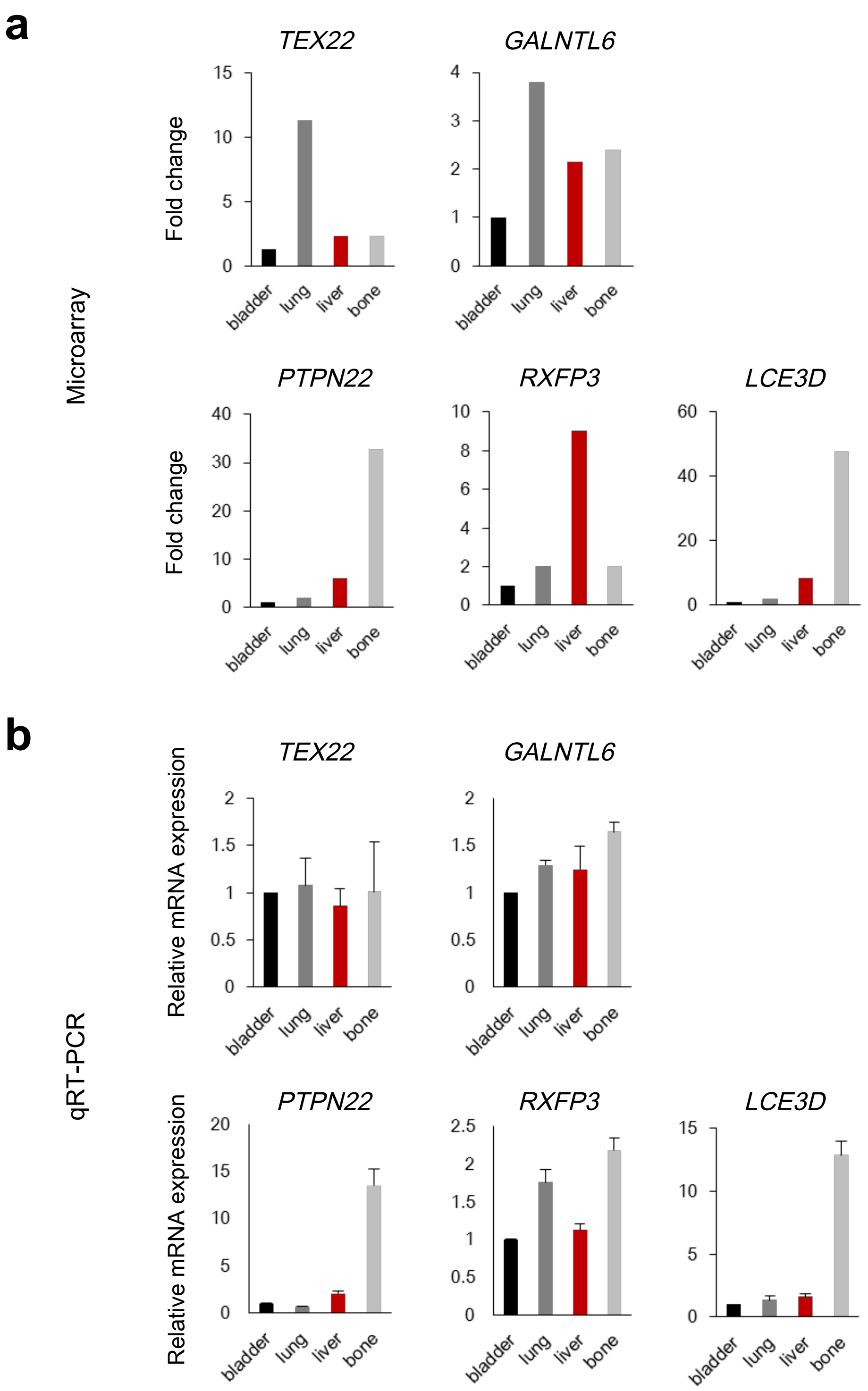

Supplementary Figure 2. Expression levels of *TEX22*, *GALNTL6*, *PTPN22*, *RXFP3*, and *LCE3D* mRNA among UM-UC-3 sublines (primary bladder, lung-, liver-, and bone-metastatic tumor cells) were examined by microarray (**a**) and real-time RT-PCR (**b**).

Supplementary Figure 2

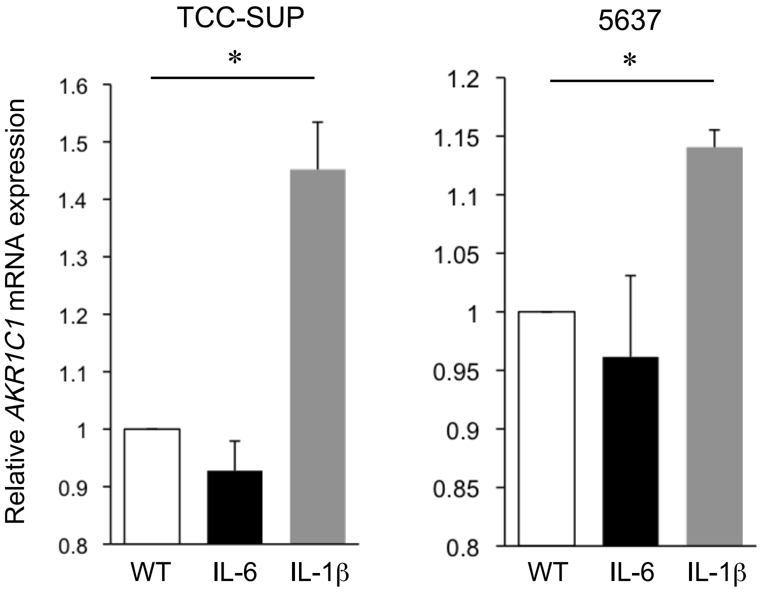

Supplementary Figure 3. TCC-SUP and 5637 bladder tumor cells were treated with IL-6 (10 ng/ml) or IL-1β (20 ng/ml). After 12 h, expression levels of *AKR1C1* mRNA were investigated by real-time RT-PCR. *P < 0.05 vs. WT.

Supplementary Figure 3

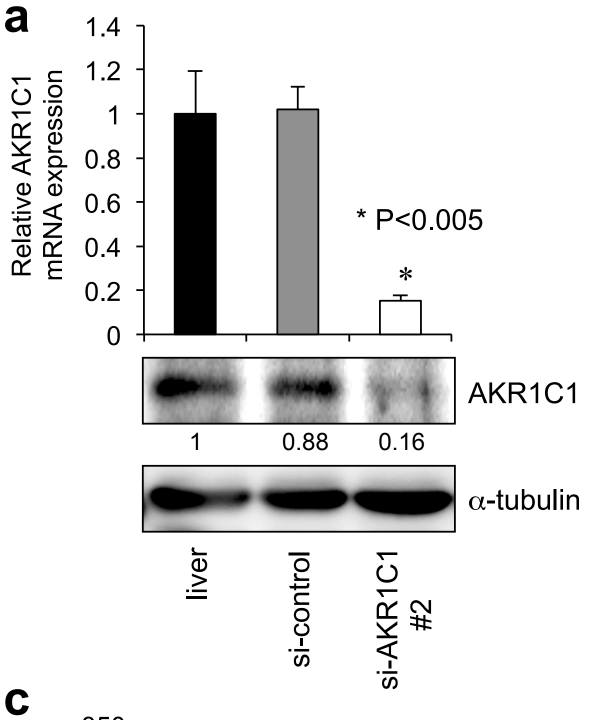

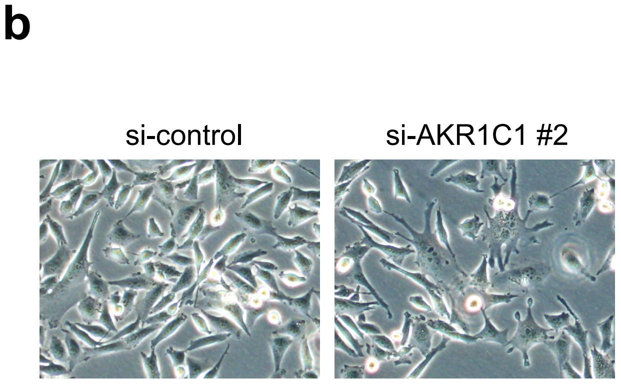

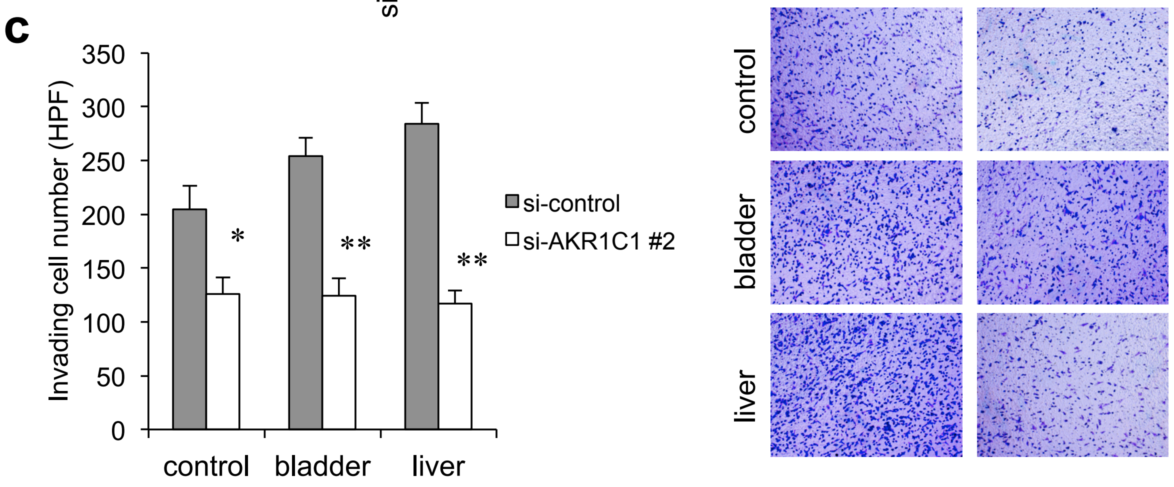

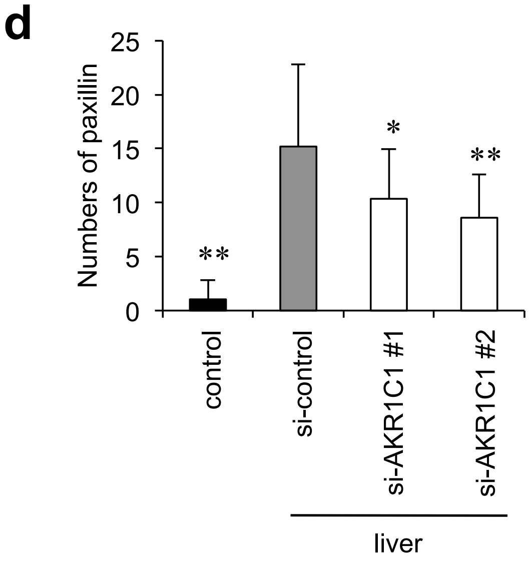

Supplementary Figure 4. (**a**) *AKR1C1* mRNA and protein expression levels in UM-UC-3-liver metastatic cells as indicated were examined by real-time qRT-PCR after 48 h (upper) and IB after 72 h (lower). (**b**) Photomicrographs of UM-UC-3-liver cells as indicated were taken under bright-field illumination at 72 h after transfection. (**c**) Effect of AKR1C1 inhibition in a Matrigel invasion assay. UM-UC-3-control, UM-UC-3-primary bladder cells, and UM-UC-3-liver-metastatic cells were treated with siRNA targeting *AKR1C1 (#2)*, which was followed by a Matrigel invasion assay. *P < 0.01 vs. si-control. **P < 0.001 vs. si-control. (**d**) Paxillin in UM-UC-3-control and UM-UC-3-liver metastatic cells as indicated was immunostained, and the numbers per cell were counted in at least more than 15 cells. *P < 0.01 vs. si-control. **P < 0.0005 vs. si-control.

Supplementary Figure 4

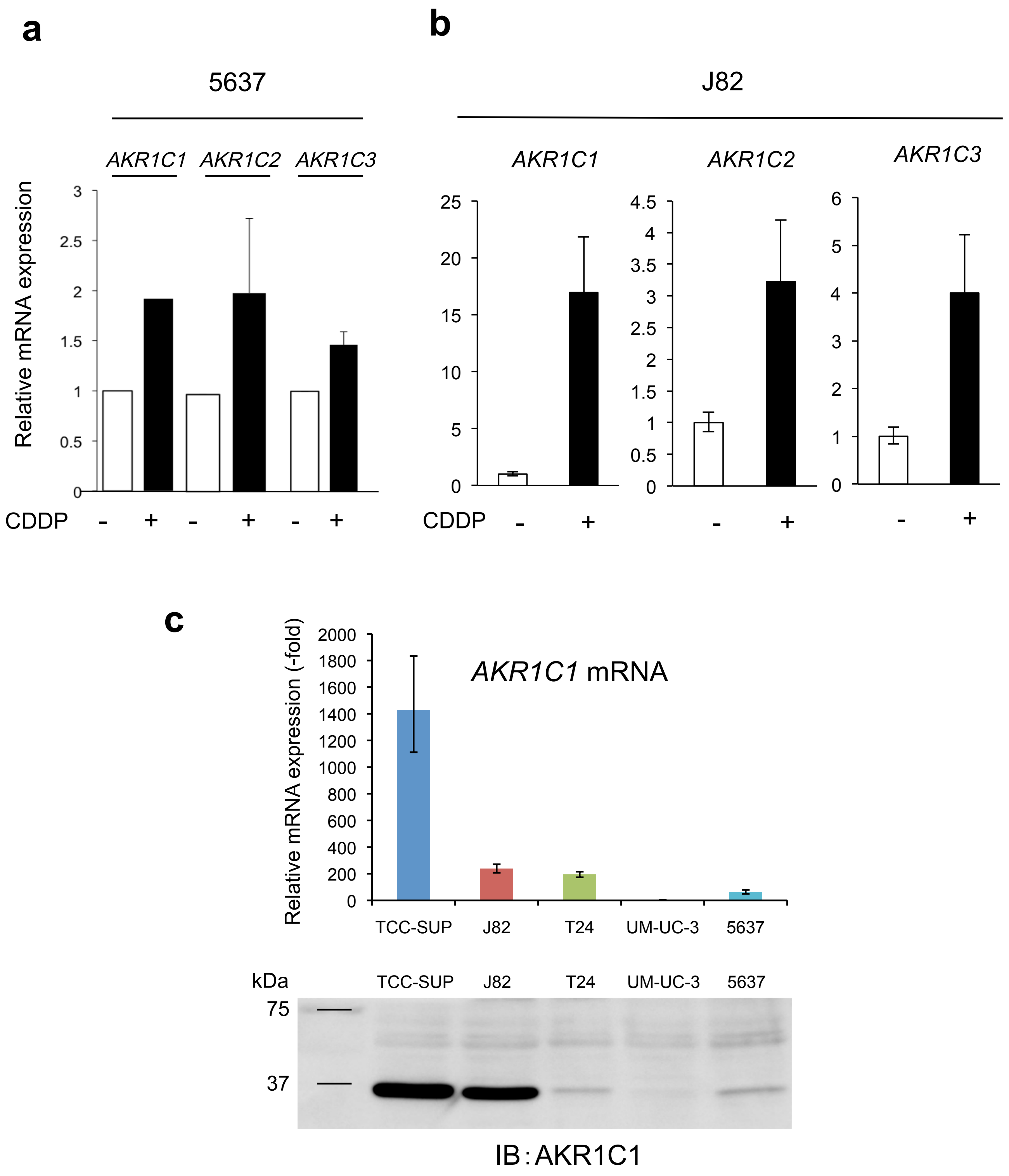

Supplementary Figure 5. 5637 (**a**) and TCC-SUP (**b**) cells were continuously treated with 1 μM and 0.3 μM cisplatin (CDDP), respectively, for 1 month, and expression levels of *AKR1C1*, *AKR1C2*, and *AKR1C3* mRNA were examined by real-time RT-PCR. (**c**) Endogenous expression levels of AKR1C1 in five bladder cancer cell lines were examined by qRT-PCR (upper) and immunoblotting (lower).

Supplementary Figure 5

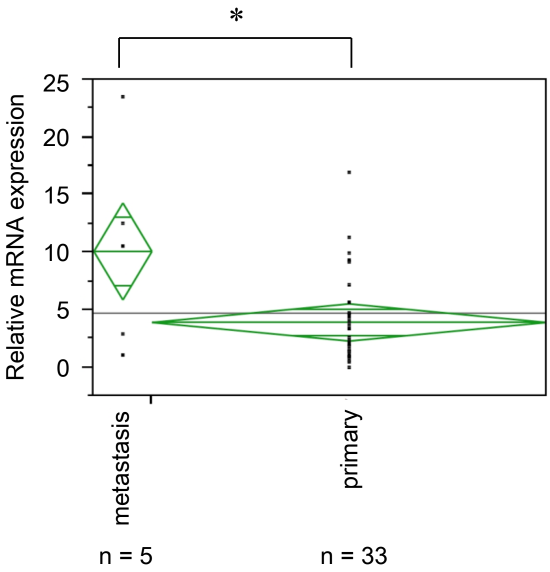

Supplementary Figure 6. AKR1C1 expression increases in human metastatic tumors. Expression levels of *AKR1C1* mRNA in human bladder cancer specimens were examined by real-time RT-PCR. Primary tumor (n = 33), metastatic tumor (n = 5). *$P < 0.05$ vs. primary.

Supplementary Figure 6

a

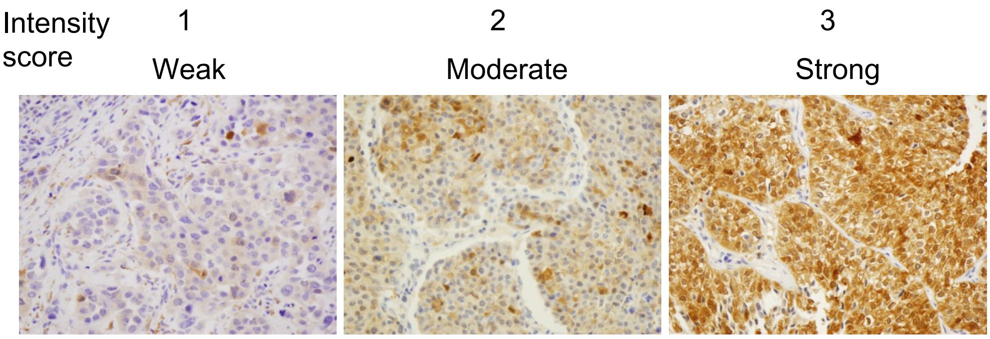

b

	Proportion	Intensity
0	0%	Negative
1+	≦10%	Weak
2+	10%～50%	Moderate
3+	≧50%	Strong

c

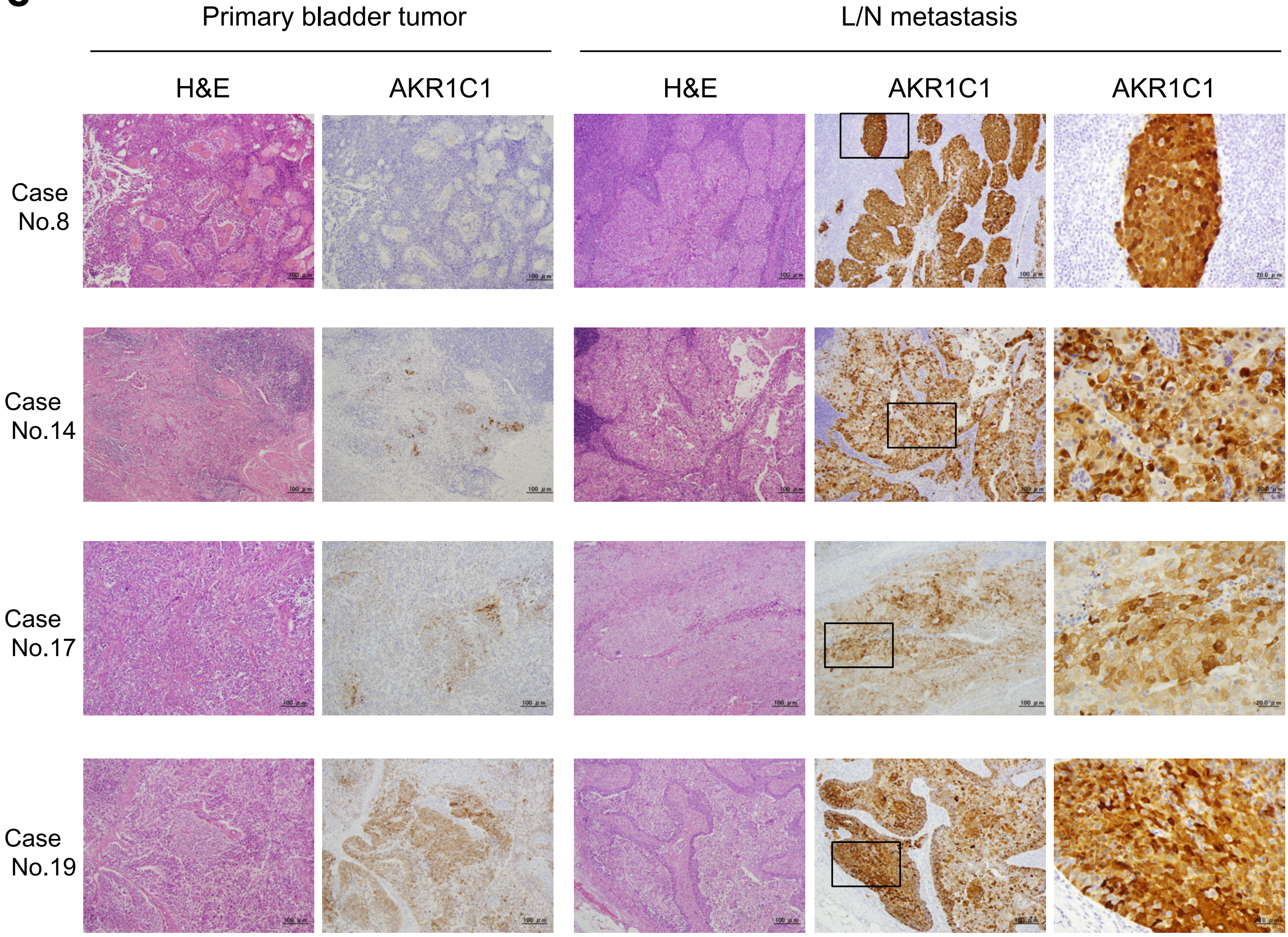

Supplementary Figure 7. (**a,b**) The expression of AKR1C1 was evaluated as the sum of intensity score (0 - 3) and proportion intensity score (0 - 3) of AKR1C1. (**c**) Primary bladder tumor and the metastatic tumor specimens were subjected to H&E and immunohistochemistry for AKR1C1.

Supplementary Figure 7

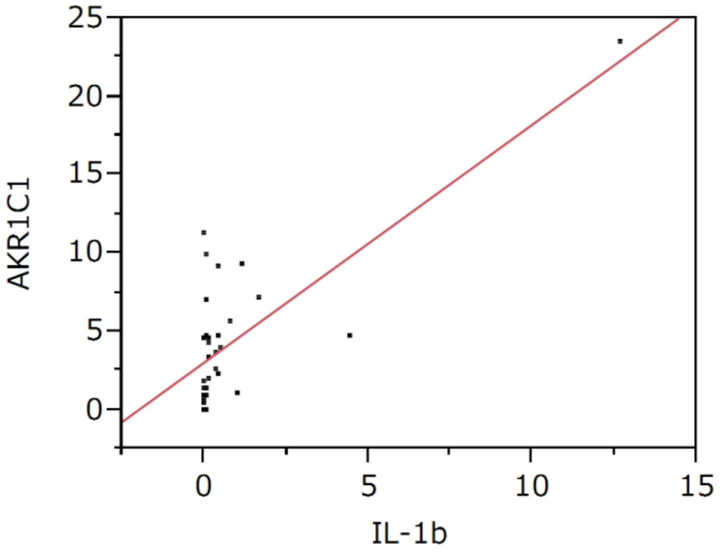

Supplementary Figure 8. Scatter plot indicates the relationships between AKR1C1 and IL-1β mRNAs. In 33 human bladder cancer specimens, expression level of *AKR1C1* mRNA was correlated with that of *IL-1β*. Spearman's $r^2 = 0.564$, $P < 0.0001$.

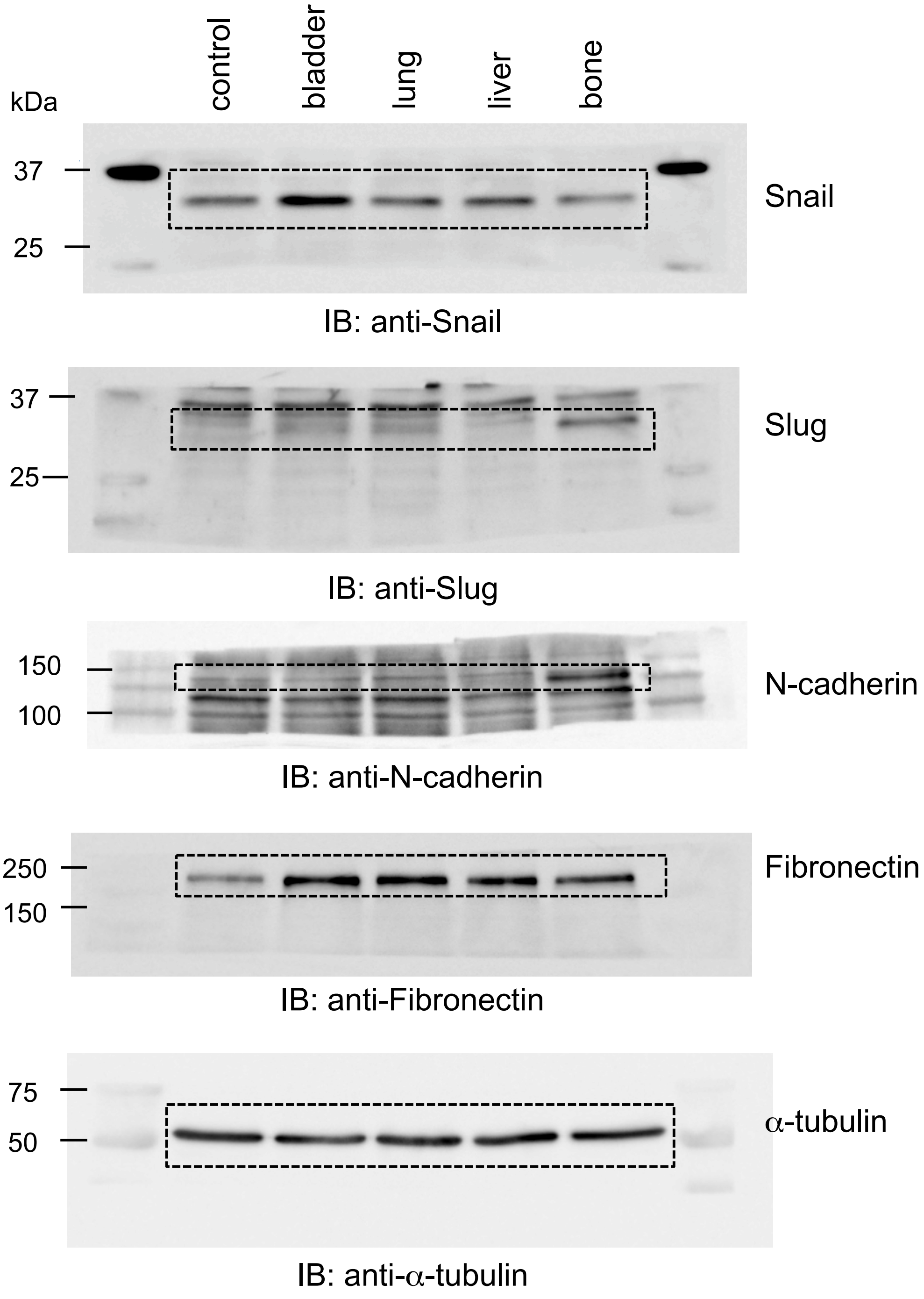

Supplementary Figure 9. Original immunoblot data for Fig. 2a.

Supplementary Figure 9

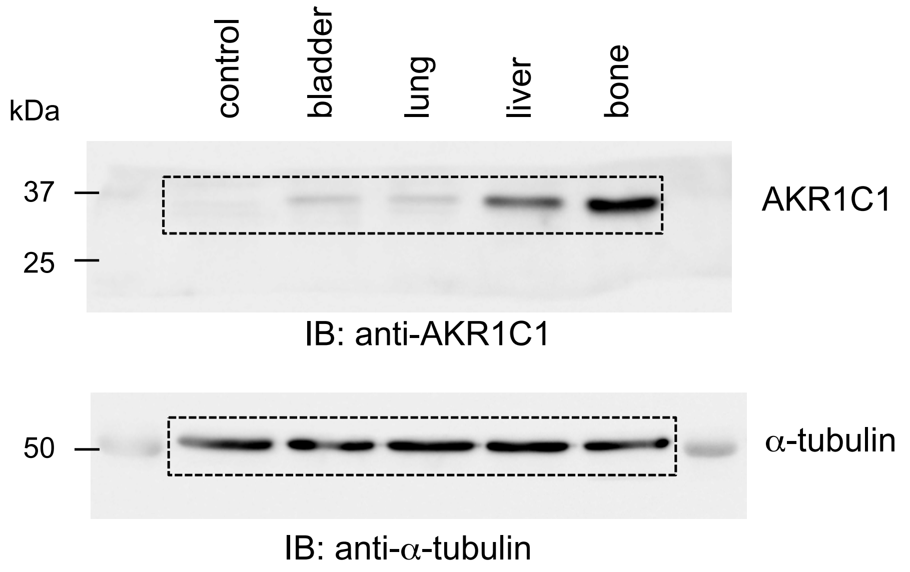

Supplementary Figure 10. Original immunoblot data for Fig. 3d.

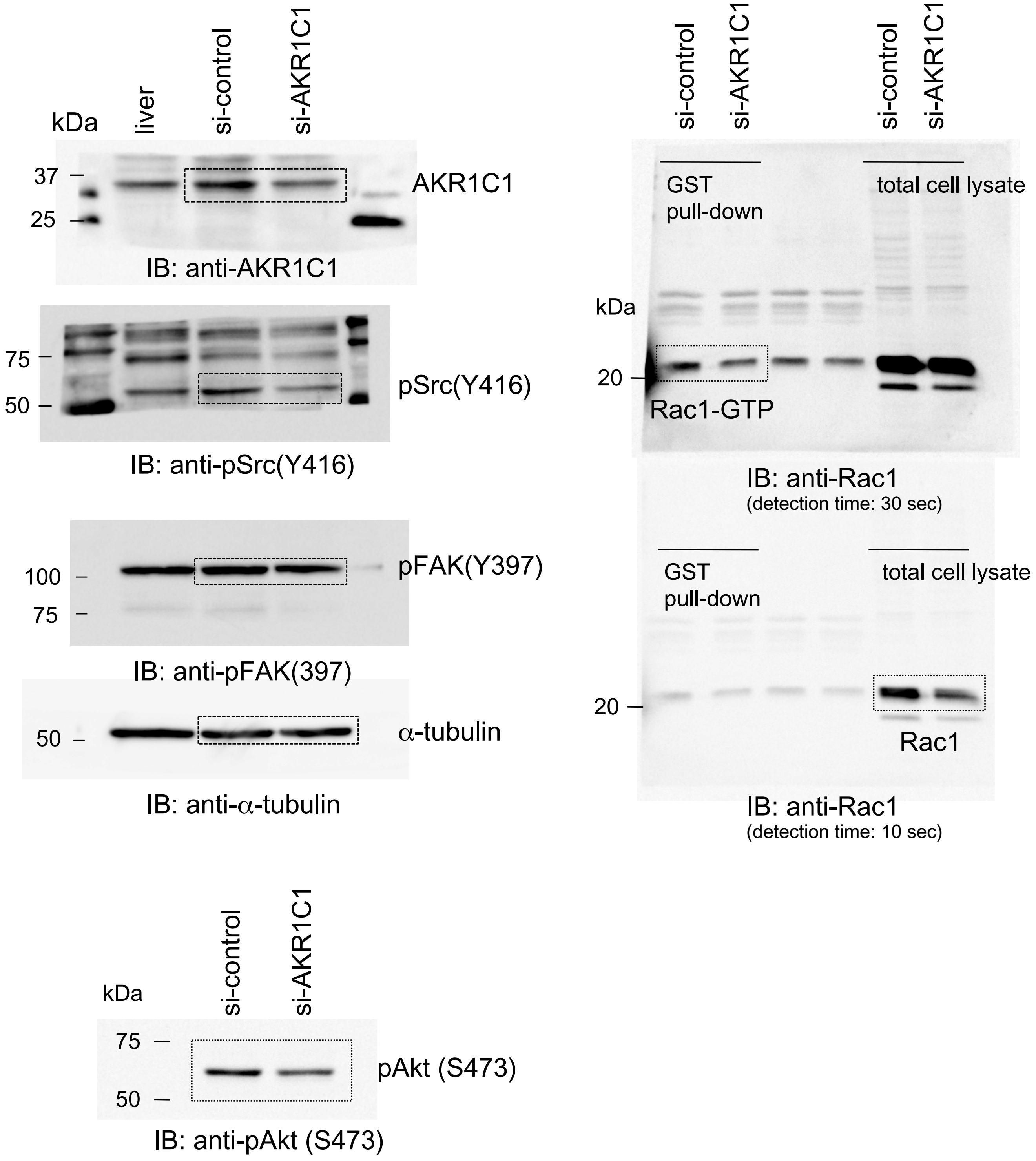

Supplementary Figure 11. Original immunoblot data for Fig. 4g.

Supplementary Figure 11

Target transcripts	Direction	Sequence
GAPDH	Forward	5' -AGCCACATCGCTCAGACAC- 3'
	Reverse	5' -GCCCAATACGACCAAATCC- 3'
MMP-2	Forward	5' -ATAACCTGGATGCCGTCGT- 3'
	Reverse	5' -AGGCACCCTTGAAGAAGTAGC- 3'
Nanog	Forward	5' -TGCAATGTCTTCTGCTGAGAT- 3'
	Reverse	5' -GTTCAGGATGTTGGAGAGTTC- 3'
CD44	Forward	5' -CTGATCATCTTGGCATCCCT- 3'
	Reverse	5' -AGCTTTTTCTTCTGCCCACA- 3'
AKR1C1	Forward	5' -CAATTGGATTATGTTGACCTCTAC- 3'
	Reverse	5' -ACTTCTCCACGGCCTCCCAC- 3'
AKR1C2	Forward	5' -AAGTAAAGCTCTAGAGGCCGT- 3'
	Reverse	5' -GCTCCTCATTATTGTAAACATGT- 3'
AKR1C3	Forward	5' -TGGAAAACTCACTGAAAAAAGC- 3'
	Reverse	5' -CATTTTCATCTGTTGGTGAAAG- 3'
IL-1β	Forward	5' -AGCTGATGGCCCTAAACAGA- 3'
	Reverse	5' -TCTTTCAACACGCAGGACAG- 3'
IL-1R1	Forward	5' -AGAGGAAAACAAACCCACAAGG- 3'
	Reverse	5' -CTGGCCGGTGACATTACAGAT- 3'
IL-6	Forward	5' -CCAGGAGAAGATTCCAAAGATGTAGCCG- 3'
	Reverse	5' -GGTTGTTTTCTGCCAGTGCCTCTTTGC- 3'
IL-6R	Forward	5' -CATTGCCATTGTTCTGAGGTTC- 3'
	Reverse	5' -AGTAGTCTGTATTGCTGATGTC- 3'
TEX22	Forward	5' -AGAAGCCACCTGACTGCACTG- 3'
	Reverse	5' -CACCTGGCTGTGTAATCTTGGAA- 3'
GALNTL6	Forward	5' -AATCTCTGTGTGGACAGCAAGCA- 3'
	Reverse	5' -ATCAAAGCAGAATTTCCGGGTATG- 3'
PTPN22	Forward	5' -AAGCCTGCAGAATCTGTTCAGTCA- 3'
	Reverse	5' -GCAGGTGTACTTGCAGCCCATA- 3'
RXFP3	Forward	5' -AGACAATTGTGCCCGTTTATTCC- 3'
	Reverse	5' -TTCTTGCCTACCAGCTCTAGATTCG- 3'
LCE3D	Forward	5' -TTGATGCATGAGTTCCCAGATAC- 3'
	Reverse	5' -TGACATCCTGGACATCAGACA- 3'

Supplementary Table 1. Primer list used for real-time RT- PCR in this study.

Background of bladder cancer cases						
Case No.	M/F	Age (year)	T stage	Grade	Histological type	Metastatic site
1	M	66	3a	2	UC	L/N
2	F	63	3a	2 > 3	UC	Lung
3	M	71	2	3	UC	Liver
4	M	80	3a	3	UC	L/N
5	M	61	3a	3	UC	L/N
6	M	73	3b	3	UC	L/N
7	M	64	3a	3	UC	L/N
8	M	72	2	3	UC	L/N
9	F	43	1	2	UC	Lung
10	F	51	3a	2	UC	Lung
11	M	57	1	3	UC	L/N
12	F	82	4	3	UC	L/N
13	M	76	3a	3	UC	L/N
14	F	66	2	3	UC	L/N
15	M	82	3a	3	UC	L/N
16	M	81	3b	3	UC	L/N
17	F	74	3a	3	UC	L/N
18	F	78	4	3	UC with adenocarcinoma	L/N
19	M	74	3a	3	UC	L/N
20	F	65	3a	3	UC	L/N
21	M	64	4	3	UC	L/N
22	M	70	4	3	UC	L/N
23	M	64	4	3	UC	L/N
24	M	80	4	3	UC	L/N
25	M	71	4	3	UC	L/N

Supplementary Table 2. Background of 25 matched-paired samples of pathologically diagnosed primary bladder and the metastatic tumors.

Supplementary Table 2
